# Evolution of a central dopamine circuit underlies adaptation of a light-evoked sensorimotor response in the blind cavefish

**DOI:** 10.1126/sciadv.adv3770

**Published:** 2026-05-22

**Authors:** Robert A. Kozol, Ally Canavan, Bernadeth Tolentino, Alex C. Keene, Johanna E. Kowalko, Erik R. Duboué

**Affiliations:** ^1^ Wilkes Honors College, Florida Atlantic University, Jupiter, FL, USA.; ^2^ Department of Biology, Texas A&M University, College Station, TX, USA.; ^3^ Department of Biological Sciences, Lehigh University, Bethlehem, PA, USA.

## Abstract

Adaptive behaviors emerge in novel environments through functional changes in neural circuits. While relationships between circuit function and behavior are well studied, how evolution shapes circuits to drive behavioral adaptation is poorly understood. The Mexican cavefish, *Astyanax mexicanus*, provides a unique genetically tractable model, with above ground eyed surface fish and multiple blind cavefish populations that have evolved in darkness. These differences in environment and vision offer a way to examine how neural circuits evolve. We examine differences in detection and behavioral responses to the nonvisual effects of light in cave and surface *A. mexicanus*. Both populations exhibit photokinesis: Surface fish become hyperactive after darkness, and cavefish after illumination. Using whole-brain functional imaging aligned to an established *Astyanax* brain atlas, we identify the caudal posterior tuberculum as key to light- and dark-induced photokinesis. Pan-neuronal GCaMP imaging shows that dark-sensitive neurons in surface fish are light-sensitive in cavefish. Light sensing depends on dopamine signaling, suggesting that a conserved dopamine circuit mediates photokinesis and highlighting *Astyanax* as a model for sensory adaptation.

## Teaser

Evolution shifts dopamine neuron responses in cavefish, reversing how light drives their behavior.

## INTRODUCTION

Behavioral responses are driven by the dynamic coordination of brain-wide neural circuits, which act in coordinated fashion to take in sensory information and elicit an appropriate behavioral response. Behaviors are finely tuned to the environment that an organism lives in, and neural circuits underlying these responses are under strict evolutionary pressure (*1*–*4*). Model systems have greatly enhanced our understanding of how neural circuits develop and modulate behavior (*5*–*7*). Studies using circuit ablations or optogenetics in wide-ranging systems from flies and fish to mammals have revealed the effect of dysregulated neural circuits and their impact on behavior. However, less is known about how neural circuits evolve to give rise to adaptive behaviors (*4*, *8*, *9*). While advances in genetic technology in model systems has made it possible to inactivate or overactivate circuits, a major impediment to understanding how behaviors evolve lies in the inability to modify neural circuits in ways that reflect naturally occurring changes. Our understanding of how evolution shapes circuits comes largely from comparing neural circuit function across closely related species that inhabit similar environments; however, large divergence times and incomplete evolutionary histories make inferring generalizable models of how neural circuits evolve difficult (*5*, *10*–*16*). Addressing this question requires a species with evolutionarily divergent forms, complete evolutionary histories, robust behavioral differences, accessibility to brain-wide neural circuits, and amenability to genetic and neuronal interrogation (*4*).

The blind Mexican tetra (*Astyanax mexicanus*) is an emerging model for understanding the evolution of neural circuits and behavior. *A. mexicanus* exists as surface-dwelling river fish and more than 30 independently evolved cave populations, which have adapted to hundreds of thousands of years of nutrient scarcity and perpetual darkness (*17*–*20*). While surface populations have retained eyes and pigment, cave populations share eye degeneration and loss of pigment, and exhibit variation in sleep, stress, feeding, and metabolism (*21*–*25*). These evolved traits can be investigated genetically by interbreeding surface and cave morphs, producing viable hybrid offspring that permits investigation of the genetic basis of trait variation, including genetic mapping (*26*–*30*). Recently, we also established transgenesis and targeted genome mutagenesis in surface and cavefish populations (*31*, *32*). This work has brought *Astyanax* into the genetic era, and has resulted in a number of stable lines such as ones that express the Ca^2^+^^ sensor GCaMP pan-neuronally (*33*). This specific transgenic line allows for functional imaging of neural responses to sensory stimuli. Together, this model is uniquely poised to further our understanding how evolution affects circuit function and behavioral adaptation.

Sensory systems provide a platform for understanding the evolution of behaviors in response to a changing environment (*34*). In zebrafish, detailed work examining the behavioral and neuronal response to changes in illumination has been particularly well studied. When fish in an illuminated background are exposed suddenly to darkness, fish become hyperactive and engage in a light searching behavior (*10*, *35*–*39*). This behavior is presumed to be an evolutionary adaptation, which allows light perception to guide fish’s behavior. Moreover, recent work has shown that such photokinesis is evolutionarily conserved in sighted fish spanning more than 200 million years of evolution (*40*, *41*). While photokinesis has not been examined in *Astyanax*, previous work has shown that nonvisual detection of changes in illumination is preserved in both surface and cave *Astyanax*. Specifically, cavefish have retained a functional pineal eye, which mediates behavioral responses to changes in light levels, such as the shadow response, where larvae exhibit an upward swimming reaction when light is dimmed (*42*). In addition, studies indicate that motor asymmetry associated with light-searching behaviors in zebrafish is at least partially retained in surface *Astyanax* and may be mediated by both visual and nonvisual pathways involving the thalamus (*43*). These findings suggest that while cavefish have lost retinal vision, they maintain alternative photoreceptive mechanisms that could play a role in behavioral adaptations to their environment.

In this study, we investigated how evolution shapes the sensorimotor processing of light stimuli in *A. mexicanus* by comparing photokinetic behavior and neural circuits across surface fish and cavefish populations. We show that cavefish exhibit a reversal in photokinetic behavior, becoming more active in light, in contrast to the dark-induced activity of surface fish. This shift likely reflects adaptations to life in perpetual darkness, involving nonvisual light detection. Whole-brain functional mapping identified circuit changes and the emergence of a novel functional cell type associated with this behavioral divergence. In addition, conserved dopaminergic neurons respond to light and contribute to the altered photokinesis, highlighting how evolutionary changes in neural function can reshape behavioral responses to environmental cues.

## RESULTS

### Identification of a fundamental sensorimotor circuit in cavefish

**Fig. 1.**
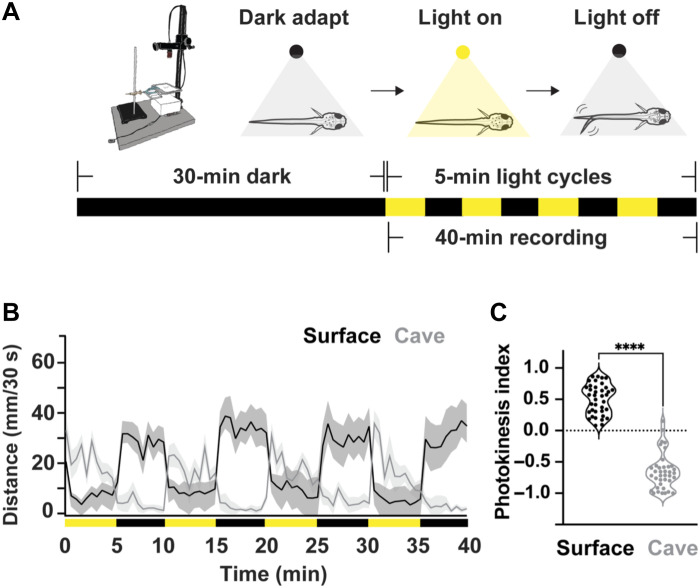
Cavefish exhibit a negative photokinesis index, whereas surface fish exhibit a positive photokinesis index. (**A**) Diagram illustrating surface fish behavior and the light cycles used in the photokinesis experiments. Surface fish exhibit increased swimming behavior in dark cycles. All fish were acclimated for 30 min in darkness (250 μl system water per well) before initiating the alternating 5-min light (yellow)/dark (black) cycles. (**B**) Line graph showing the behavioral patterns of elevated swimming activity behavior for cavefish (yellow bars indicate lights on) and surface fish (black bars indicate lights off). (**C**) Photokinesis index box plots quantifying relationship between increased activity and light cycle (positive indicates light off, negative indicates light on). Statistical significance represents output from a Tukey multiple comparisons corrected one-way ANOVA. **P < 0.01, ****P < 0.0001.

Zebrafish and other sighted teleosts exposed to sudden periods of darkness become hyperactive and display a “light-searching” behavior (*36*, *43*, *44*). To test these responses in surface and cave *Astyanax*, we devised a similar assay to record locomotor behavior under alternating periods of 5-min lights on followed by 5-min lights off (Fig. 1A). Surface fish showed baseline locomotor patterns under periods of illumination, and elevated locomotor activity during dark periods. Conversely, we found that cavefish had baseline locomotion in darkness and enhanced locomotion when the lights were turned on (Fig. 1B), suggesting a change in their preference for illumination. To quantify how strongly each fish responded to changes in illumination, we devised a metric, the photokinesis index, which compares activity changes between light transitions. The photokinesis index is calculated as the difference between activity changes when the lights turn off versus when they turn on, normalized by the total activity change across both transitions (Fig. 1A; see Materials and Methods). A positive photokinesis index indicates the fish increased swimming behavior when the lights were turned off, whereas a negative photokinesis index suggests fish became active when lights were turned on. We found that both zebrafish (*Danio rerio*) and *Astyanax* surface fish exhibit positive photokinesis indices consistent with their increased swimming behavior during the lights off phase (Fig. 1C and fig. S1), whereas cavefish exhibit negative photokinesis indices, suggesting that they increase swimming during the lights on phase (Fig. 1, B and C, and fig. S1). These results reveal that individuals from this population of cavefish that adapted to perpetual darkness conditions have altered photokinetic responses.

### Whole-brain mapping reveals differences in neural activity underlying photokinesis

**Fig. 2.**
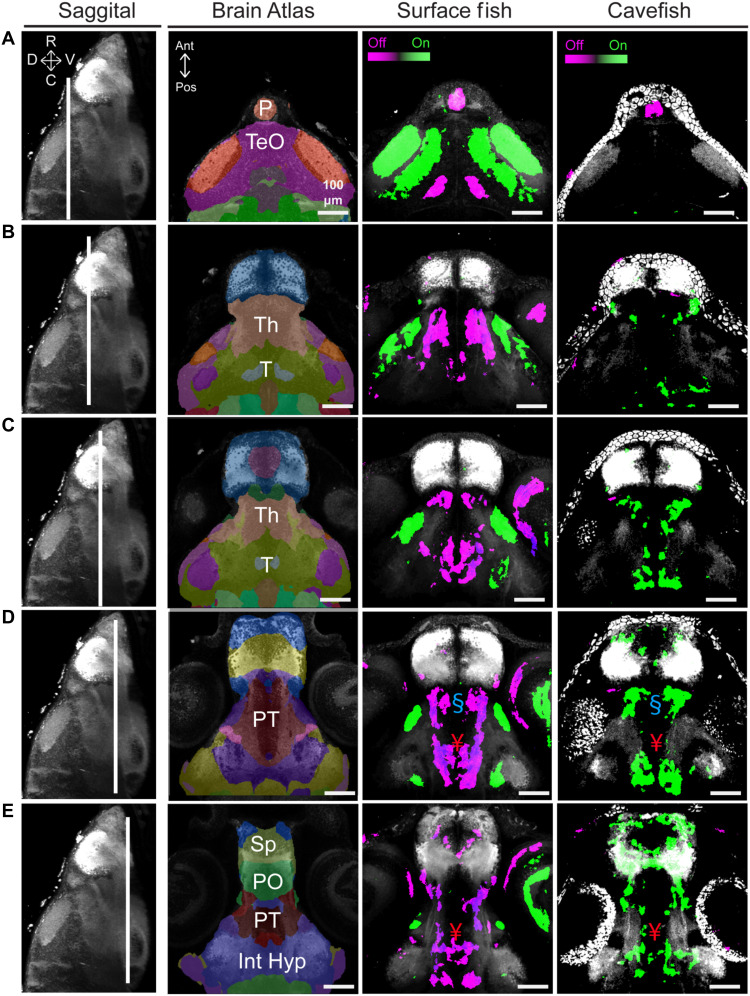
Brain mapping identifies overlap and variation of neural activity in the ventral forebrain between surface fish and cavefish. The first column displays a sagittal maximum projection of the reference atlas (white) through the middle of the brain, with directional arrows representing dorsal (D), ventral (V), rostral (R), and caudal (C). White lines indicate the optical section. The second column provides an optical section through the *Astyanax* brain atlas and tERK reference brain. The last two columns contain pERK neural activity maps displaying regions of increased pERK immunostaining during lights off (magenta) or lights on (green) in surface fish and cavefish. Rows represent dorsal to ventral optical sections through the (**A**) pineal (P) and optic tectum (TeO), (**B** and **C**) thalamus (Th) and tegmentum (T), and last, (**D** and **E**) the subpallium (SP), preoptic region (PO), posterior tuberculum (PT), and intermediate hypothalamus (Int Hyp). White regions represent reference anatomy from the Astyanax brain atlas used for registration. Sample sizes, surface fish (*n* = 18) and cavefish (*n* = 20). § and ¥ denote significance in the preoptic and posterior tuberculum, respectively.

A functional sensory circuit typically contains neurons that receive input, neurons that process that input, and neurons that produce output (*16*, *45*). We sought to identify the neurons that are active during light and dark periods in both cave and surface fish forms. To characterize this sensorimotor circuit in surface fish and cavefish, we performed phospho–extracellular signal–regulated kinase (pERK) mitogen-activated protein kinase (MAP) mapping of the entire *Astyanax* brain (*46*–*48*), comparing neural activity between lights-on and lights-off transitions within surface fish and cavefish populations. pERK is used as a proxy for neural activity, exhibiting peak phosphorylation within 3 to 5 min of neuronal firing (*48*). Groups of fish were split into two groups, those exposed to either lights off or lights on for 5 min, stained immunohistochemically for total ERK (tERK) and pERK, and then compared within a population (e.g., surface fish or cavefish). For light-receptive regions, we found increased neural activity in the optic tectum of surface fish when the light was turned on, while the pineal body showed increased neural activity when the lights were turned off, recapitulating photokinesis activity maps in zebrafish (*49*) (Fig. 2, A and B). Conversely, cavefish did not exhibit increased activity in light-receptive regions under either condition but did show increased activity in the pineal body during lights off (Fig. 2, A and B). Both morphs exhibited pineal activation following changes in illumination, and quantitative comparisons revealed no significant differences across conditions. These data are consistent with shared pineal responsiveness and divergent downstream processing. While surface fish retain a functional retina that detects light transitions, our data suggest that the pineal contributes to the nonvisual light-evoked locomotor response observed in both morphs. Because of its standing role as a light-sensing organ (*50*, *51*), the pineal is the likely anatomical locus sensing changes in illumination in both cavefish and surface fish.

We next asked what brain region could be critical to deep brain light processing. We reasoned that the region processing light would show enhanced neural activity in surface fish when the lights turned off, whereas the same area would increase neural activity in cavefish when the lights turned on. We found two anatomical areas in the ventral forebrain that matched this expectation. When registered to the *Astyanax* brain atlas (*47*), we found that this area mapped to the preoptic region of the hypothalamus (blue symbol) and the posterior tuberculum (red symbol; Fig. 2, D and E). Together, this suggests a neural circuit involving the pineal as a light-sensing organ, and the hypothalamus and posterior tuberculum as processing centers. Moreover, the lack of significant differences in pERK staining in the pineal, along with opposite activity profiles for the central sensory processor suggests that environmental pressure is acting on the central processor of light and not a primary light-sensing organ.

### Analysis of hybrid fish suggests photokinesis is a heritable trait

**Fig. 3.**
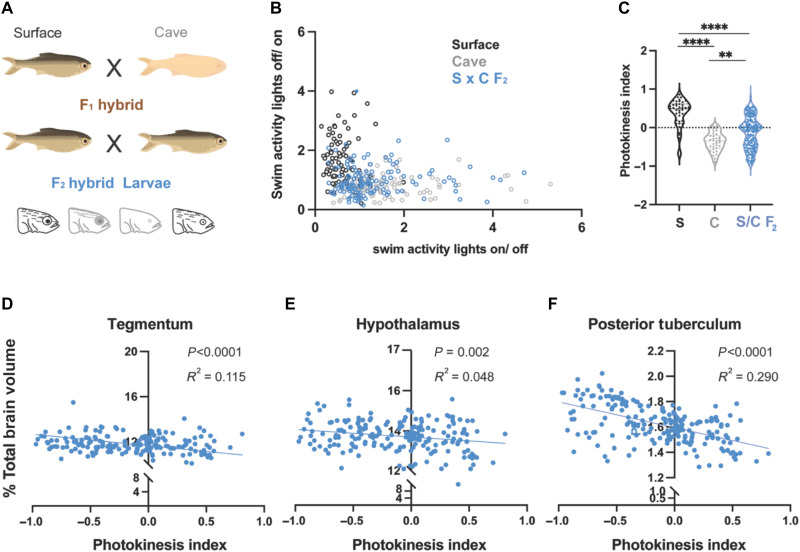
Photokinesis behavior in F_2_ hybrids is highly variable and photokinesis indices are negatively correlated to brain region size. (**A**) Diagram illustrating the crossing scheme for producing F_2_ hybrid larvae to correlate behavior to biological traits. (**B**) Scatter plot displaying the relationship between light-on and light-off transition states. Surface fish are grouped along the *y* axis, cavefish along the *x* axis, and F_2_ hybrids are scattered across both grandparental populations. Sample sizes: surface fish (*n* = 42), cavefish (*n* = 34), and surface-to-cave F_2_ hybrids (*n* = 199). (**C**) Photokinesis index violin plots exhibiting the range of behaviors of F_2_ hybrids, from positive to negative photokinesis values. Scatter plots of brain region volume against photokinesis behavior for (**D**) tegmentum, (**E**) hypothalamus, and (**F**) posterior tuberculum. Pearson’s correlation was used for all correlation plots.

A strength of the *Astyanax* system is the ability to cross different populations to one another, and study the genetics that underlie complex traits. We first crossed a pure surface fish with a cavefish isolated from the Pachón cave to produce a brood of F_1_ hybrid fish. Compared to pure surface and cavefish, F_1_ exhibited an intermediate photokinetic phenotype (fig. S2, brown circles). We then crossed F_1_ fish with one another to produce a population of F_2_ hybrid fish, and subjected these larvae to our photokinesis assay. We found varying levels of photokinesis in F_2_ hybrids with some progeny reacting more similarly to surface fish, and others reacted similarly to cavefish (Fig. 3, B and C, F_2_ hybrids are blue circles). When examined as a population, F_2_ hybrid fish spanned the entire range of surface to cave. The photokinetic mean of F_2_ hybrid fish was near zero (−0.0355), with a much higher variability than either pure breeding population (photokinetic mean of surface fish = 0.3754, photokinetic mean of cavefish = −0.3687, and photokinetic mean of F_2_ hybrids = −0.0355). These data are consistent with a genetically encoded trait. These results suggest that photokinesis differences are driven by complex, multilocus allelic variation between wild surface fish and cavefish populations.

### Hybrid brain-wide analysis reveals correlations between photokinesis and volume of the posterior tuberculum

Because of the established relationship between functional variation and anatomical differences within a population (*52*, *53*), we next asked whether changes in neuroanatomical volume were associated with the observed differences in photokinesis between surface and cavefish. To determine whether brain region volume correlated with differences between surface fish and cavefish photokinesis behavior, we assessed light-evoked activity in F_2_ hybrid fish, and then examined correlations between this behavior and the volume of every known neuroanatomical region using a recently published *Astyanax* brain atlas (*47*). Each individual hybrid larva was behaviorally tested and scored in our photokinesis assay, stained for brain-wide neuroanatomy using an ERK antibody, and computationally analyzed for regional brain volume. We then performed a parametric correlation analysis between neuroanatomical volume and photokinesis indices (Fig. 3 and fig. S3). Surface-cave F_2_ larvae exhibited negative correlations between photokinesis indices and volume of the tegmentum, hypothalamus, and posterior tuberculum (Fig. 3, D and E), supporting a role for anatomical evolution of the ventral forebrain in modulating these behaviors. In contrast, no other brain region showed a significant correlation between photokinesis indices and regional volume (fig. S3). These results suggest that light and dark photokinesis is likely influenced, in part, by anatomical variation of the ventral forebrain in *Astyanax* populations.

### Neurons in the posterior tuberculum exhibit novel off-to-on responses in cavefish

**Fig. 4.**
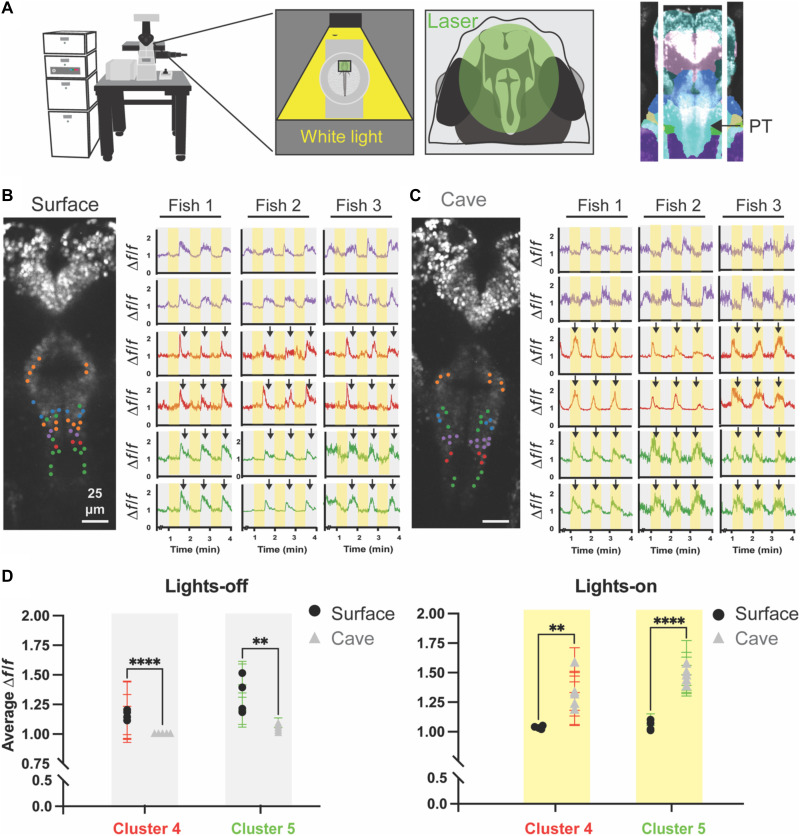
Clusters of neurons in the posterior tuberculum of cavefish exhibit light-on tuning in comparison to light-off tuning in surface fish. (**A**) Diagram illustrating the experimental setup of light-stimulated, agarose-embedded surface and cavefish GCaMP6s larvae. Projection inset shows the segmented region analyzed in the live imaging dataset. Surface fish (**B**) and cavefish (**C**) time-series clusters based on stimulus tuning, dark cycles (gray stripes) or light cycles (yellow stripes). Peaks are noted by black arrows for cluster 4 (red) and cluster 5 (green). Coronal projections represent analyzed regions and display color coded neuronal clusters. (**D**) Box plots comparing average delta *f*/*f* (Δ*f*/*f*) for lights-off and lights-on conditions of clusters 4 and 5 in the caudal posterior tuberculum. Sample size, surface fish (*n* = 4) and cavefish (*n* = 5). Statistical significance represents *t* test comparisons between populations for each cluster according to light conditions, **P < 0.01, ***P < 0.001, ****P < 0.0001.

Functional in vivo imaging provides a powerful tool to examine the relationship between neural activity and behavior. We sought to determine whether spatiotemporal neural activity of cells within the posterior tuberculum is differentially modulated by light in surface and cavefish. We developed stable transgenic *Astyanax* lines that express the genetically encoded calcium indicator GCaMP6s in nearly every neuron of the brain [*Tg(_zfish_elavl3:H2B-GCaMP6s)*] (*54*, *55*). Larvae were paralyzed in 5 μl of bungarotoxin, mounted in a drop of 2% low-melt agarose, placed under a confocal microscope, and subjected to 30-s alternating periods of lights on and lights off (Fig. 4A). We found that the posterior tuberculum contained light-responsive clusters of neurons (Fig. 4, B and C). Each cluster in surface fish showed a light-off tuned response, and the patterns of activity among clusters were stable across different larvae (Fig. 4B, black arrows in gray stripes). By contrast, while clusters of neurons in the medial portion of cavefish brain were also light-off responsive, clusters in the caudal portion of the posterior tuberculum exhibited light-on responses, consistent with photokinesis in response to illumination in this morph (Fig. 4C, black arrows in yellow stripes). To quantify variation across morphs, we calculated calcium transient averages for clusters 4 and 5 and compared them across light conditions (Fig. 4D). Last, in both surface fish and cavefish, rostral regions exhibited no clear light-evoked activity (fig. S4). Therefore, the posterior tuberculum is unique in its differential response to light between surface and cavefish populations.

### Photokinetic neurons in the posterior tuberculum are dopaminergic

**Fig. 5.**
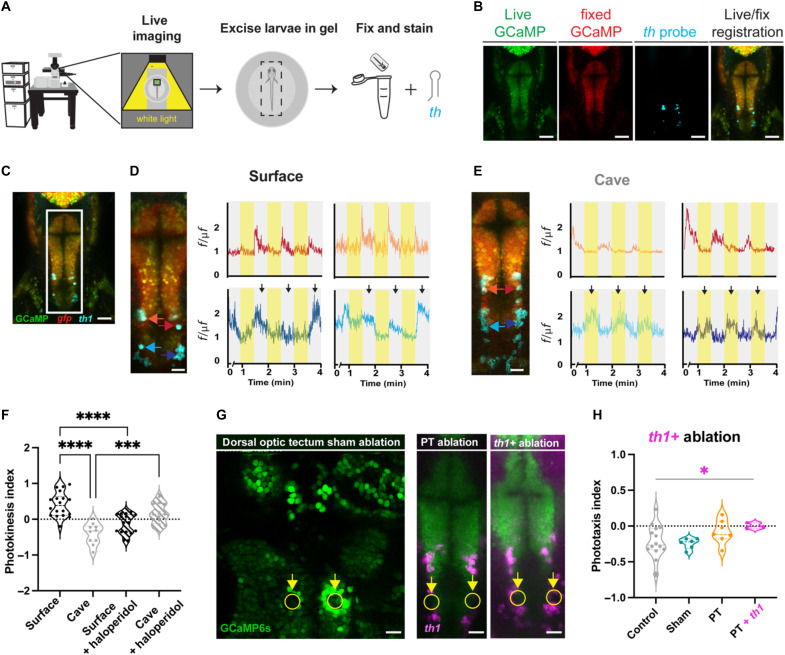
Light-tuned posterior tuberculum neurons are dopaminergic and photokinesis is extinguished by dopamine antagonism or physical ablation. (**A**) Pipeline for live-to-fixed imaging. Neural activity is recorded, larvae are fixed in agarose and stained with RNA probes. (**B**) Stained larvae are then imaged and registered to a maximum projection of the live stack. (**C** to **E**) Medial projection of the posterior tuberculum with colored dots and arrows corresponding to neural activity traces for surface fish (D) and cavefish (E). Image resolutions are (B and C) 512 × 512, zoom 1.2; and (D and E) 512 × 128, zoom 2.4. (**F**) Violin plot showing photokinesis indices for surface fish, cavefish, surface exposed to haloperidol, and cavefish exposed to haloperidol. Sample sizes, surface fish (*n* = 18), cavefish (*n* = 10), surface fish + haloperidol (*n* = 15), and cavefish + haloperidol (*n* = 12). (**G**) Examples of two-photon sham, posterior tuberculum (PT) and *tyrosine hydroxylase* (*th*)–positive ablations. (**H**) Photokinesis indices following ablation experiments. Sample sizes, cavefish (*n* = 14), cavefish sham ablations (*n* = 6), cavefish PT ablations (*n* = 7), and cavefish PT + *th* ablations (*n* = 3). Scale bars, 512 × 512 = 50 μm and 512 × 128 = 20 μm. Statistical significance represents a Tukey multiple comparisons corrected one-way ANOVA. ***P < 0.001, ****P < 0.0001.

The posterior tuberculum comprises many different cell types, yet one of the most conserved are the dopaminergic neurons of the descending diencephalic system. These cells are thought to be analogous to the A11 dopaminergic cells in mammals and modulate diverse internal states including stress, mood, and reward (*56*, *57*). To assess whether light-evoking cells in *Astyanax* were dopaminergic, we used a unique method that combines live GCaMP imaging with subsequent RNA in situ hybridization to molecularly identify neurons that are active in light or dark conditions. In brief, following live GCaMP imaging, larvae were fixed in agarose to preserve the brain’s position and hybridization chain reaction (HCR) in situ hybridization was performed to visualize expression of a dopaminergic marker, *tyrosine hydroxylase* (*th*; Fig. 5, A and B). Live-to-fixed registration of GCaMP channels revealed that caudal neurons of the posterior tuberculum with opposite dark to light stimulus tuning express *th* (Fig. 5, C to E). These data suggest that the descending diencephalic system could be integrating light signals and sending the information to hindbrain motor areas, and this conserved set of neurons may be responsible for differences in light preference between surface and cavefish.

### A11 dopaminergic neurons in the posterior tuberculum modulate light-evoked locomotion

To functionally test if dopamine plays a role in photokinesis behavior, we exposed surface fish and cavefish to the typical antipsychotic dopaminergic inhibitor, haloperidol, and then tested larvae for photokinesis behavior. We found that haloperidol abolishes photokinesis behavior in both surface fish and cavefish (Fig. 5F and tables S21 and S22), further supporting the notion that dopaminergic neurons play a role in photokinesis behavior. While these data reveal that dopamine is necessary for photokinesis, the widespread nature of dopamine in the brain means that pharmacological treatments alone cannot confirm the A11 descending diencephalic system as the necessary neurons for photokinesis behavior. To confirm that the A11 neurons were indeed required for light preference, we used a multiphoton laser to physically and specifically ablate cavefish neurons in the caudal region of the posterior tuberculum and then determined whether these specific neurons affect photokinesis behavior (fig. S5). Experimental ablations were targeted to the caudal portion of the tuberculum, with sham ablations targeted to dorsal visual regions of the brain as a control (Fig. 5G and fig. S5). In contrast to control groups, ablations to the posterior tuberculum reduced photokinesis behavior, while ablation of *th*-expressing neurons provided the largest change in behavior (Fig. 5H). Together, our molecular maps, coupled with dopamine antagonism and physical ablations in cavefish, suggest that dopaminergic neurons of the posterior tuberculum play a key role in behavioral variation across populations of *Astyanax*.

## DISCUSSION

Neural circuit evolution underlies the functional variation necessary for behavioral adaptation in a new environment. Behavioral adaptation has long been linked to anatomical and physiological changes in the brain, yet little is known about how neural circuits driving these behaviors evolve due to the complexity and lack of functional tools needed to study these relationships. This study tackles this impediment with a system that couples an evolutionary nonmodel vertebrate with cellular-resolution functional tools. Our discovery that cavefish have evolved light-evoked photokinesis allowed us to ask what brain regions are affected and which neuronal subgroups could contribute to behavioral variation. The fact that all previously studied eyed fish exhibit dark photokinesis and that only cavefish exhibit a light photokinesis suggests that the development of light photokinesis is reflective of evolving in a cave environment (*36*, *43*). This study also continues to expand our knowledge of the neurological basis of trait evolution in cavefish and more generally for sensory systems in vertebrates.

### Light photokinesis as an adaptive behavior for avoiding light

Light photokinesis has developed convergently in cave fauna across the globe, suggesting that light photokinesis or photophobia is a cave-adapted behavior. In these ecosystems, animals are adapted to the light-deprived nature of caves and become less adept at surviving in above-ground territories (*58*). Past studies have found that cave-adapted insects and fish exhibit agitation and increased activity when stimulated by light and tend to retreat to dark conditions, behaviors termed photophobia or photokinesis (*59*–*65*). These studies suggest that light-evoked activity is likely an adaptation for staying away from cave entrances and karst windows (sunken cave roofs), which could in turn place these organisms in an environment they are not conditioned for. Photokinesis would therefore reduce predator interactions or keep the cave-adapted organisms from venturing toward surface environments (*59*, *61*–*65*). This includes a study where light photokinesis was observed in cavefish that maintained an intact pineal, supporting our photokinesis brain mapping that shows increased activity for surface and cave morphs during photokinesis (*42*, *66*). Therefore, our experiments provide functional insight into ethologically observed behavior and suggest that forebrain circuits underlying photokinesis are evolving to functionally change light-evoked behavior.

### Surface-to-cave hybrid behavior suggests that photokinesis is genetically inherited

Surface-to-cave hybridization provides a functional genetic test to determine whether phenotypes are genetically encoded (*28*, *67*). A trait is considered to be genetically encoded when an F_2_ hybrid population’s traits span the range of pure surface and pure cave traits. We observed a wide range of photokinetic responses in surface-to-cave F_2_ hybrids, from surface-like dark activating to cave-like light activating, providing evidence that development of light or dark photokinesis is genetically encoded (*68*, *69*). Cave light photokinesis could be a consequence of fixed genetic variants that affect sensorimotor integration during larval development.

### An existing neuronal subgroup may explain photokinesis in cavefish

While our research suggests that tubercular neurons play a role in photokinetic behavioral variation, we have yet to confirm whether this variation is due to a novel subset of neurons or a functional change in a group of neurons with shared ontogeny. Sensory circuits can be modified by several different mechanisms, including the loss or gain of a receptor, changes in accessory structures of a neuron, and inclusion or exclusion of interneuron subgroups (*3*, *16*, *70*). Photokinetic circuit function could be caused by the evolution of a neo-functional cell type, where the regulatory machinery is reprogrammed to produce alternative functions in comparison to an ancestral cell type (*71*, *72*). Alternatively, a major shift in opsin wavelength-tuning expression of varying opsins across populations could provide a receptive/integrative mechanism for producing light photokinesis (*73*–*75*). However, we have yet to look at the genetic and developmental mechanisms driving variation in the posterior tuberculum, including genes of interest that could be causing differences in photokinesis. Last, we present evidence that dopamine plays a functional role in cavefish photokinesis behavior; however, we have not functionally tested the role of A11 dopaminergic neurons in surface fish dark photokinesis behavior. Future work looking at the development and function of these circuits is needed to clarify the evolutionary mechanism producing this variation in behavior.

### Dopamine linked to evolution of the photokinetic variation in cavefish

Dopaminergic neurons play integral roles in sensorimotor integration and behavioral state changes (*10*, *56*, *57*, *76*, *77*). We identified A11 dopaminergic neurons in cavefish that are light-responsive in comparison to dark-responsive neurons in surface fish. A11 dopaminergic neurons have been shown to be highly conserved, with studies in fish, rodents, and primates sharing the same developmental gene regulatory network (*56*, *57*, *76*). Previous work has shown that A11 dopaminergic neurons are one of the primary sources of descending dopamine to the spinal cord in mammals, and that their activation leads to descending motor signals and locomotion (*56*, *76*). In zebrafish, associations exist between neuronal activity in A11 dopaminergic neurons and several stimuli. This included subclasses that were reactive during spontaneous behavior, mechanosensory stimuli, and visual stimuli (*78*). Specifically, dorsal medial dopaminergic neurons are active during spontaneous behavior and visually stimulated tuberal hypothalamic neurons were reactive to a forward moving stimulus. The authors speculate that tuberal hypothalamic neurons may modulate reticulospinal neurons during swimming or during the optomotor response (*78*). Our ablation results show that the posterior tuberculum and A11 dopaminergic neurons could provide a regional mechanism for modulating phototaxis; however, more functional work is needed to clarify functional roles in surface and cave populations.

### A potential mechanism for photokinesis variation

Variation in monoamine metabolism may provide a mechanism for cavefish photokinetic response. Monoamine metabolism is necessary to produce and regulate the neuromodulators dopamine, serotonin, and noradrenaline (*79*, *80*). Previous work examining the monoamine synthesis system in *Astyanax* revealed that cavefish have a mutation in the *monoamine oxidase* (*mao*) gene that contributes to high levels of dopamine, serotonin, and noradrenaline (*81*, *82*). Alternatively, a mutation in the *oculocutaneous albinism 2* (*oca2*) gene that underlies a pigment synthesis pathway, may also lead to high levels of dopamine (*67*, *83*). These changes in monoamine metabolism were hypothesized to play a role in the “behavioral syndrome” that is observed in cavefish, including changes in sleep, stress, and feeding (*82*). We believe that variation in monoamine levels could play a role in light photokinesis. However, further genetic and developmental studies are needed to determine the true cause of light photokinesis in cavefish.

In conclusion, we find that cavefish provide a unique model for studying the evolution of sensory processing and sensorimotor behavior. Our work highlights the importance of neural circuit evolution for processing environmental stimuli and functional adaptation to a novel environment. This work supports a light sensory processing role for dopaminergic neurons of the posterior tuberculum, while future work on sensory processing in the forebrain of cavefish will lead to a more comprehensive understanding of the mechanisms underlying the evolution of sensory systems.

## MATERIALS AND METHODS

### Fish maintenance and husbandry

Zebrafish and Mexican tetras were housed in the Florida Atlantic University’s zebrafish and Mexican tetra core facilities. Larval fish were maintained at 28°C (zebrafish) and 23°C (Mexican tetra) in system water and exposed to a 14:10 hour circadian light:dark cycle. Zebrafish and Mexican tetras were cared for in accordance with NIH guidelines and all experiments were approved by the Florida Atlantic University Institutional Care and Use Committee, protocol numbers A 17-21 and A22-44. *A. mexicanus* surface fish lines used for this study were all lab bred and include surface fish from the Texas population and transgenic Texas surface fish *Tg(_zfish_elavl3:H2B-GCaMP6s*). Cavefish lines used for this study include Pachón cavefish and Pachón *Tg(_zfish_elavl3:H2B-GCaMP6s*). GCaMP transgenic lines were produced in a previous study (*31*, *33*). Surface fish were crossed to Pachón to generate F_1_ hybrids, while F_1_ hybrid offspring were intercrossed to produce surface-to-cave F_2_ hybrids. Surface-to-Pachón F_1_ hybrids were generated by crossing a single breeding pair consisting of a surface fish and Pachón cavefish. One sibling surface-to-Pachón F_1_ mating pair was then intercrossed to generate surface-to-Pachón F_2_ hybrid larvae for all hybrid experiments.

### Photokinetic behavioral experiments

Photokinesis experiments were performed using a custom designed behavior chamber equipped with a light box, illuminated from below by white light (380 to 780 nm) and infrared (850 nm) light-emitting diodes. Videos were collected at 25 fps with 1280 × 960 resolution using a Basler acA1300-60gm camera fitted with a 12-mm Megapixel lens or a Basler acA1300-200um (Edmund Scientific Co., Barrington, NJ, #33978) fitted with a 16-mm C-series NIR-VIS lens (Edmund Scientific Co., #67714) and a UV-Visual cutoff filter (Edmund Scientific Co., #65796). All photokinesis experiments were run using an American National Standards Institute and the Society for Biomolecular Sciences compatible 96-well microtiter plate. Each well contained a single 6 days postfertilization (dpf) larva and each larva was tested once. Data were collected and analyzed using the EthoVision XT software versions 11.5 and 14 (Noldus Inc., Leesburg, VA). *Astyanax* fish were acclimated to the observation chamber at 23°C. All fish were acclimated for 30 min in darkness (250 μl system water per well) before initiating the alternating 5-min light/dark cycles.

Fish movement was continuously captured via IR illuminated video recording. All behavioral experiments were recorded between 11 a.m. and 3 p.m., with three to five independent trials per condition.

### Statistics

Ethovision-generated raw data were binned in 30-s intervals and sorted into light cycles with averages generated for values across light pulses using custom MATLAB (Mathworks, Natick, MA) scripts. Average lights on or off transition periods (e.g., 30 s before light change and 30 s after light change) were then used to calculate the photokinetic index. The photokinetic index was calculated as

$$\text{PI}=\frac{∆t_{\text{on}\to \text{off}}-∆t_{\text{off}\to \text{on}}}{∆t_{\text{on}\to \text{off}}+∆t_{\text{off}\to \text{on}}}$$

then imported into PRISM version 8.1.2 (GraphPad Software, Dotmatics, Boston, MA). Behavior was assessed for normality and then statistically assessed using *t* tests for wildtype and one-way analysis of variance (ANOVAs) for hybrid comparisons. Tukey *P* value corrections were applied following statistical significance for ANOVAs.

### Brain-wide phospho-ERK mapping

ERK phosphorylation methods were carried out using a previously published analysis for comparing ERK phosphorylation [MAP mapping (*48*)]. Larvae exposed to lights on for cavefish and lights off for surface fish were used as the experimental condition, with the larvae from the opposite light condition being selected as the control state. *Z*-stacks were imaged on a Nikon A1R multiphoton microscope, using a water immersion 25×, numerical aperture (NA) 1.1 objective.

### Automated segmentation and brain region measurements

To segment and measure subregions of the brain, we used a previously published analytical method (*47*) that registers each larval brain to the *Astyanax* brain atlas, segments the brain using advanced normalization tools (*84*), and computes regional brain volume for all brain regions in the *Astyanax* atlas (CobraZ) (*85*). Last, a simple linear regression was applied to determine whether brain region volume correlated with photokinesis indices.

### Genetically encoded calcium imaging

Surface fish and Pachón cavefish GCaMP6s stable transgenic embryos were screened for green fluorescent protein expression and grown to 6 dpf. Larvae were incubated in bungarotoxin (1 mg/ml) dissolved in system water for 1 min to induce paralysis before being moved to a recovery bowl with 250 ml of system water. Following 5 min of system water recovery, larvae were embedded in 2% low–melting point agarose dissolved in system water and placed on the confocal stage to acclimate for 15 min. Following acclimation, larvae were imaged for 4 min, 1 min of no light following laser initiation, followed by 30-s intervals of light on and light off.

### Live GCaMP light on and off response analysis

Time series stacks were analyzed using mesmerize, a live-imaging analysis suite that uses the program CaImAn for stimulus tuning and neuronal clustering (*86*). Time series stacks and regions of interests saved in FIJI were imported, along with stimulus periods, then motion corrected and saved as a dataframe. Dataframes were run through the raw data normalization, stimulus tuning, linkage matrix, and hierarchical clustering functions (max_cluster = 5), to cluster neurons according to light and dark responsiveness. These clusters were then mapped using the plot dataframe function and saved as a png.

### Molecular mapping of fixed to live images

Larvae were screened and GCaMP was imaged as described previously (*33*). Following imaging, larvae were euthanized in tricaine, excised in agarose, and fixed in 4% formaldehyde overnight at 4°C. Agarose blocks were then washed 3× with 1× phosphate-buffered saline and stained using a previously published Hybridization Chain Reaction (HCR) in situ hybridization protocol (*47*). HCR probes against surface fish and cavefish *th* mRNA were generated and used to probe the posterior tuberculum. Blocks of stained agarose embedded larvae were then mounted in an imaging well using a drop of 2% low–melting point agarose and imaged on a Nikon A1R confocal microscope with a 20× long-working distance 0.9-NA water-dipping objective. Images were then registered to live imaged slices using a previously published method (*47*).

### Dopamine antagonist exposure

A previously published protocol was used to expose larval fish to the dopaminergic antagonist haloperidol (*87*). Five days postfertilization larval fish were exposed to 500 nM haloperidol dissolved in 0.1% dimethyl sulfoxide (DMSO) (experimental) or 0.1% DMSO (control) overnight. The following day 6 dpf larvae were assayed for photokinesis behavior and analyzed as previously stated in the behavioral and statistical methods sections.

### Two-photon physical ablation

Larvae were anesthetized using tricaine (25 mg/liter), embedded in 2% agarose, and then subject to two-photon laser ablation. Larvae brains were ablated using a Zeiss 20×, 1.0 NA, W Plan-Apochromat, water immersion (catalog no. 421452-9880). The following laser settings were used to physically ablate 10-μm^3^ bilateral circles: wavelength of 880 nm, 80% power, 8.3 mW, laser engaged for 5 s, and 20× water-immersion objective. Following ablation, larvae were extracted from agarose and allowed to recover overnight. The following day, larvae were assayed for photokinesis behavior and analyzed as previously stated in the sections “Photokinetic behavioral experiments” and “Statistics.”

## Supplementary Materials

### The PDF file includes:

Figs. S1 to S5 Legend for supplementary Excel file

### Other Supplementary Material for this manuscript includes the following:

Supplementary Excel file
