# Supplementary material for: Evolution of a central dopamine circuit underlies adaptation of a light-evoked sensorimotor response in the blind cavefish
Source: Sci Adv. 2026 May 22;12(21):eadv3770. doi: 10.1126/sciadv.adv3770 (PMC13196759; doi:10.1126/sciadv.adv3770)
Supplement: Supplementary file 1 — Figs. S1 to S5 Legend for supplementary Excel file [file sciadv.adv3770_sm.pdf]

Science Advances
AAAS

Supplementary Materials for

Evolution of a central dopamine circuit underlies adaptation of a light-evoked sensorimotor response in the blind cavefish

Robert A. Kozol *et al.*

Corresponding author: Erik R. Duboué, eduboue@FAU.edu

Sci. Adv. **12**, eadv3770 (2026)
DOI: 10.1126/sciadv.adv3770

The PDF file includes:

Figs. S1 to S5
Legend for supplementary Excel file

Other Supplementary Material for this manuscript includes the following:

Supplementary Excel file

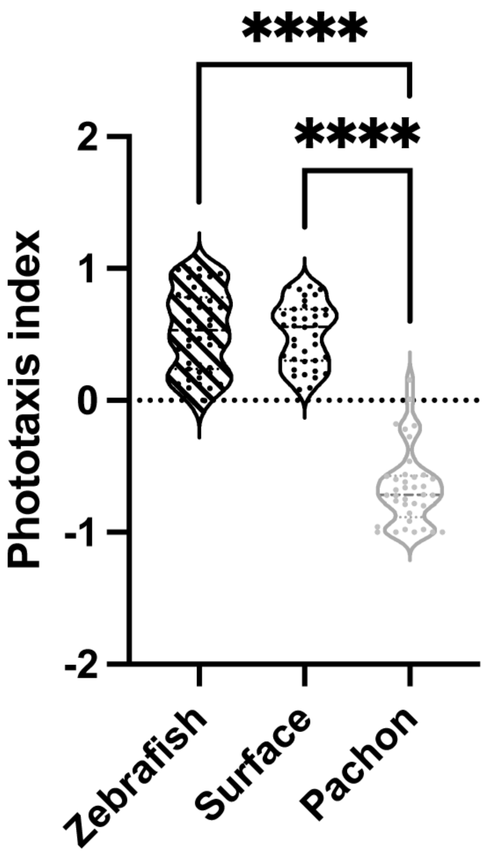

Supplementary Figure S1. Phototaxis comparisons between Danio and Astyanax. Zebrafish and surface fish exhibit positive photokinesis indices, while cavefish exhibit negative photokinesis indices. **** indicates p>0.0001

Supplementary Figure S1

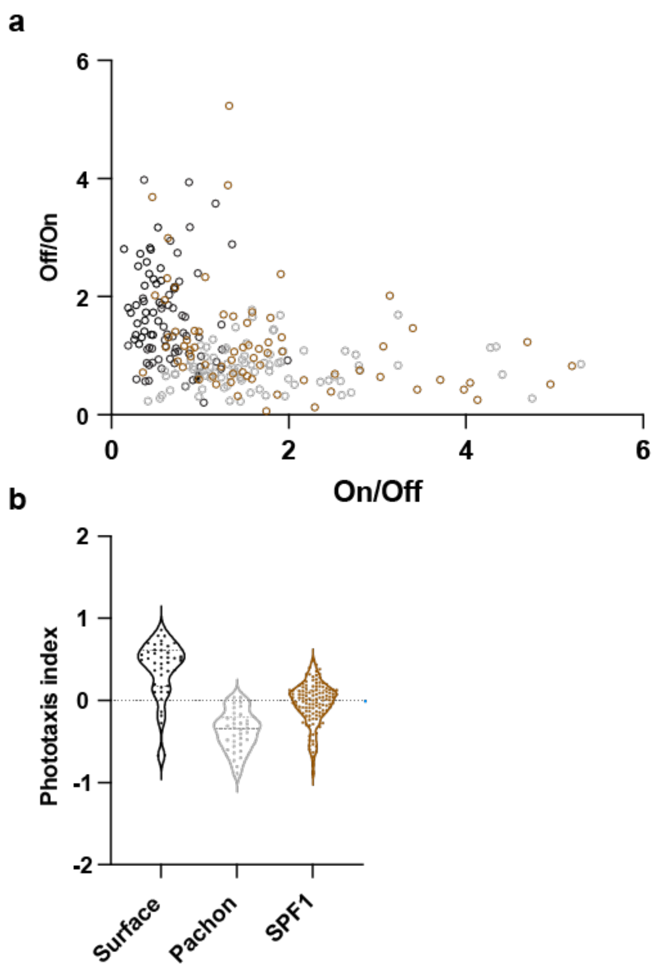

Supplementary Figure S2. Surface to cave F1 hybrids exhibit a wide range of indices that suggests photokinesis is genetically inherited. (a) Scatter plot for each larval tested. X-axis are values for activity during when the lights are turned on (off -> on), whereas y-axis are values for activity when lights are turned off (on -> off). (b) Phototaxis indices for Surface, Pachon, and Surface Pachon F1 hybrids. Black is Surface, grey is Pachón, and orange are F1 hybrid fish.

Supplementary Figure S2

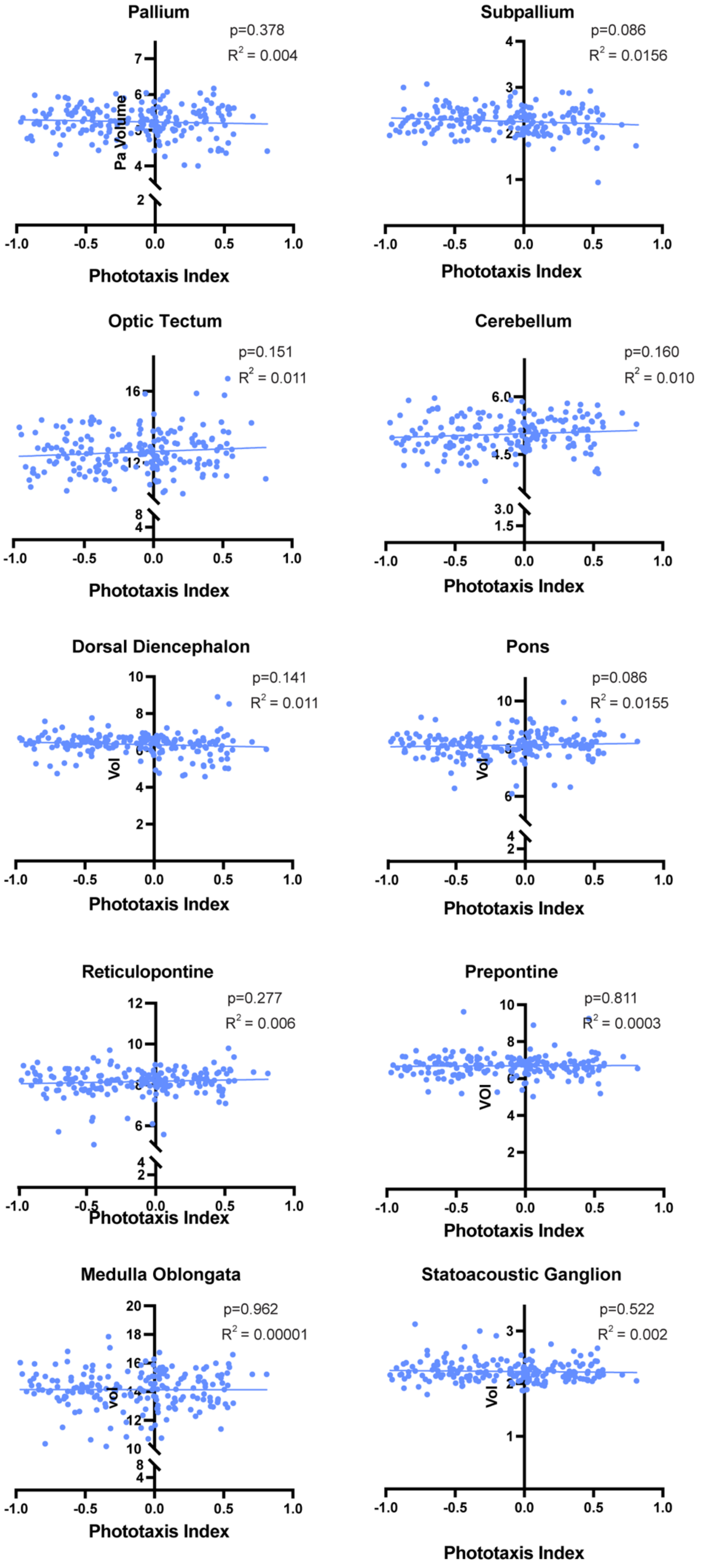
Pallium
p=0.378
R^2 = 0.004
Pa Volume
Phototaxis Index
Subpallium
p=0.086
R^2 = 0.0156
Phototaxis Index
Optic Tectum
p=0.151
R^2 = 0.011
Phototaxis Index
Cerebellum
p=0.160
R^2 = 0.010
Phototaxis Index
Dorsal Diencephalon
p=0.141
R^2 = 0.011
Vol
Phototaxis Index
Pons
p=0.086
R^2 = 0.0155
Vol
Phototaxis Index
Reticulopontine
p=0.277
R^2 = 0.006
Phototaxis Index
Prepontine
p=0.811
R^2 = 0.0003
VOl
Phototaxis Index
Medulla Oblongata
p=0.962
R^2 = 0.00001
Vol
Phototaxis Index
Statoacoustic Ganglion
p=0.522
R^2 = 0.002
Vol
Phototaxis Index

Supplementary Figure S3. Correlations between brain regions and behavior. Brain regions exhibiting no correlation between brain region volume and behavior. Scatter plots showing the correlations between neuroanatomical volume and phototaxis indices.

Supplementary Figure S3

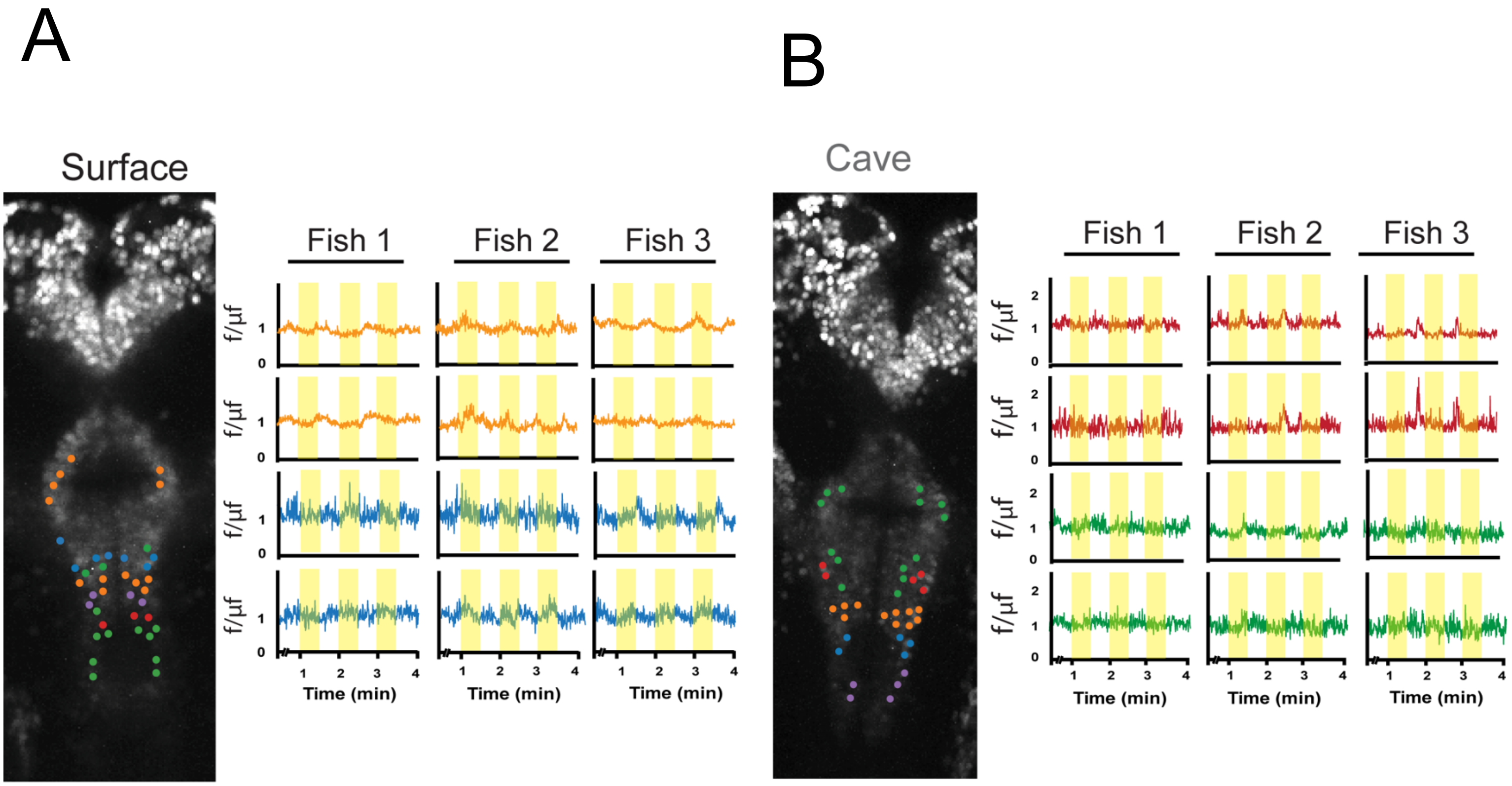

Supplementary Figure S4. Rostral clusters of the posterior tuberculum do not exhibit light stimulus tuning for either lights-on or lights-off. An example of a surface fish (A) and Pachon cavefish (B) optical slice with each functional cluster showing in colored dots. Adjacent to the image are representative values of spontaneous activity for three separate fish.

Supplementary Figure S4

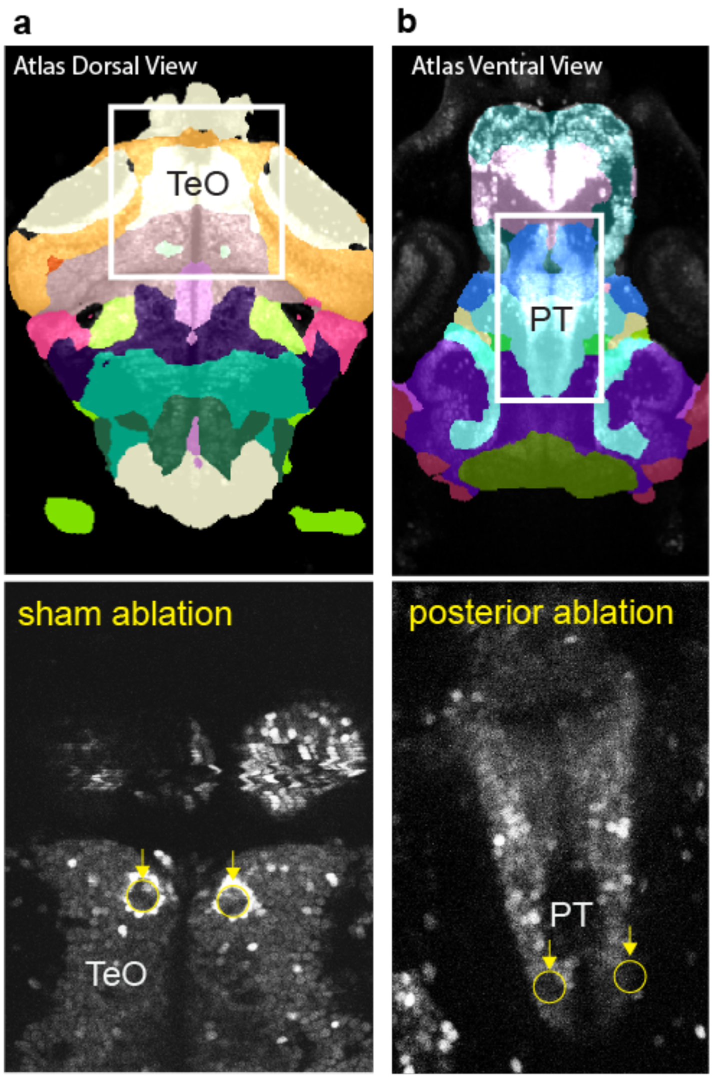

Supplementary Figure S5. Examples of ablations in dorsal visual regions and the ventral posterior tuberculum. (A) Optical slice from our Astyanax brain atlas showing the site of the sham ablation (top) and a representative ablation (bottom). (B) Optical slice from our Astyanax brain atlas showing the site of the PT ablation (top) and a representative ablation.

Supplementary Figure S5

Supplementary Excel File: Complete Statistical Dataset. The full statistical dataset underlying all figures and analyses in the study. It includes raw test outputs, summary metrics, effect sizes, sample sizes, and p-values for every behavioral, neural activity, and molecular comparison described in the manuscript.

Supplementary Excel File
